# Incidental Moyamoya Disease in an Elderly Patient Presenting With Acute Ischemic Pontine Stroke

**DOI:** 10.7759/cureus.36198

**Published:** 2023-03-15

**Authors:** Hadi Abou-El-Hassan, Haroon Azhand, Melvin Kantono, Ankur Bhagat, David Karp, Taylor Baranski, Tehmina Salahuddin, Farbod Farmand

**Affiliations:** 1 Neurology, Arrowhead Regional Medical Center, Colton, USA; 2 Internal Medicine, Arrowhead Regional Medical Center, Colton, USA; 3 Internal Medicine, California University of Science and Medicine, Colton, USA

**Keywords:** cerebral angiogram, antiplatelet therapy, stroke, moyamoya angiopathy, moyamoya disease (mmd)

## Abstract

Moyamoya disease (MMD) is a rare occlusive cerebrovascular disease that is characterized by progressive stenosis of the terminal portion of the internal carotid artery and its main branches with compensatory development of dilated and fragile collateral vasculature at the base of the brain. MMD has a bimodal age distribution commonly affecting children and adults, whereas onset in the elderly population is a rare occurrence. Here, we present a case of a 78-year-old patient of Indonesian descent who was incidentally found to have moyamoya arteriopathy after presenting with acute ischemic stroke in the left pons. The patient underwent diagnostic cerebral angiogram that showed right middle cerebral artery stenosis with pathognomonic collateral moyamoya vessels. The patient was discharged on antiplatelet therapy. We report a rare case of an elderly patient with MMD. The role of medical or surgical management in asymptomatic MMD in elderly patients remains largely unknown.

## Introduction

Moyamoya disease (MMD) is a chronic, occlusive cerebrovascular disease characterized by steno-occlusive changes of the large intracranial arteries with compensatory development of collateral vasculature known as moyamoya vessels (MMVs) [1]. In the United States, the incidence of moyamoya arteriopathy is approximately 0.57 per 100,000, with an average age of 31.6 years old, 72% of which are females [2]. While there is no known definite etiology, the high incidence of MMD among the East Asian populations strongly suggests a genetic predisposition [3]. When collateral MMVs exist with causative diseases or associated conditions such as sickle cell disease or systemic lupus erythematosus, patients are classified as having moyamoya syndrome (MMS) [4]. The most common presentations of patients with MMD are ischemic stroke and intracranial hemorrhage [5].

MMD exhibits a bimodal age distribution, usually affecting adults between 45-49 years old as well as children between five to nine years old [6]. MMD in the elderly population is extremely rare, with one of the oldest reported patients being 82 years old [7]. Here, we present a rare case of an elderly patient who was found to have MMD Suzuki stage 3 after presenting with an acute ischemic stroke in the left pons.

## Case presentation

**Figure 1:**
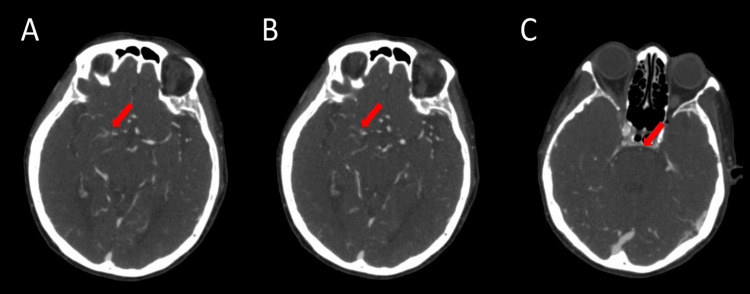
CT angiography of the head (A, B) Two consecutive axial slices showing focal occlusion of the right middle cerebral artery at the proximal M1 segment with prominent collateral flow into its peripheral branches. (C) Axial sequence showing stenosis of the basilar artery. CT: computed tomography. Red arrow indicates the pathology of interest.

**Figure 2:**
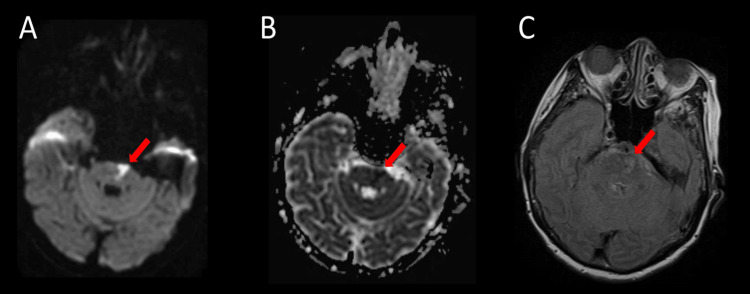
MRI of the brain without contrast (A) DWI and (B) ADC axial sequences showing a focus of restricted diffusion in the left pons. (C) FLAIR hyperintensity in the left pons. MRI: magnetic resonance imaging, DWI: diffusion-weighted imaging, ADC: apparent diffusion coefficient, FLAIR: fluid attenuated inversion recovery. Red arrow indicates the pathology of interest.

**Figure 3:**
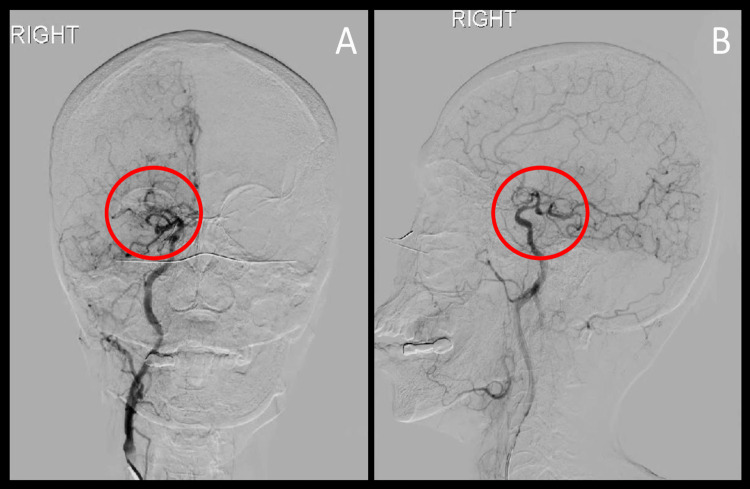
Cerebral digital subtraction angiogram (A) Coronal and (B) sagittal views of the right internal carotid artery showing right MCA stenosis with hyperplasia of lenticulostriate vessels. MCA: middle cerebral artery. Red circle indicates the pathology of interest.

A 78-year-old female of Indonesian descent with no known past medical history presented to the emergency department with a two-week history of right-sided weakness and mild slurred speech. Upon examination, blood pressure was 130/77 mmHg, heart rate was 98 beats per minute, oxygen saturation was 98% on room air, body mass index was 19.4 kg/m2, and blood glucose was 170 mg/dL. A physical exam revealed 4/5 motor strength in the right distal and proximal upper and lower extremities with no other neurological deficits. The patient's pre-admission modified Rankin Scale (mRS) score was 0. The patient denied any alcohol consumption, cigarette smoking, or illicit drug use. Family history was non-contributory, and an EKG showed normal sinus rhythm. Laboratory studies done on admission were unremarkable except for hyperlipidemia (Table 1), for which the patient was taking atorvastatin 10 mg daily. A non-contrast head CT showed no acute intracranial abnormality. Thrombolysis was not given. CT angiography of the head showed chronic-appearing focal occlusion of the right middle cerebral artery (MCA) at the proximal M1 segment with prominent collateral flow into the peripheral branches of the right MCA (Figure 1A, 1B) as well as basilar artery stenosis (Figure 1C). Transthoracic echocardiogram showed a left ventricular ejection fraction of 70-75% without any right-to-left shunting. MRI of the brain without contrast showed a focus of restricted diffusion in the left pons (Figure 2). The patient was started on dual antiplatelet therapy with aspirin 81 mg once daily and clopidogrel 75 mg once daily, as well as atorvastatin 40 mg once daily due to suspected intracranial atherosclerosis. Cerebral digital subtraction angiogram showed right MCA stenosis with hyperplasia of lenticulostriate vessels (Figure 3). The patient was evaluated by our physical therapist, occupational therapist, and speech therapist, who recommended acute rehabilitation. The patient was then discharged to an acute rehabilitation facility on antiplatelet therapy. The patient spent three weeks at the acute rehabilitation facility, after which she was discharged home.

**Table 1:** Initial laboratory studies demonstrating hyperlipidemia μL: microliter, g: gram, dL: deciliter, fL: femtoliter, mEq: milliequivalent, L: liter, mmol: millimole, mg: milligram, ng: nanogram, mL: milliliter, mIU: milli-international units, pg: picogram, BUN: blood urea nitrogen, TSH: thyroid-stimulating hormone, HbA1c: hemoglobin A1C, HDL: high-density lipoprotein, LDL: low-density lipoprotein.

Laboratory test	Measured value	Reference range
White blood cells (cells/μL)	6,000	4,300-11,100
Hemoglobin (g/dL)	12.6	12.1-15.1
Hematocrit (%)	38	36-46
Platelet (cells/μL)	223,000	120,000-360,000
Mean corpuscular volume (fL)	90	80-100
Sodium (mEq/L)	135	135-145
Potassium (mEq/L)	4.0	3.5-5.5
Chloride (mEq/L)	98	98-110
Bicarbonate (mmol/L)	25	24-34
BUN (mg/dL)	17	8-20
Creatinine (mg/dL)	0.7	0.5-1.5
Troponin (ng/mL)	< 0.30	0.0-0.30
TSH (mIU/L)	2.13	0.35-5.5
HbA1c (%)	6.4	< 5.7
Vitamin B-12 (pg/mL)	1,676	250-1100
Folate (ng/mL)	> 20	2.8-25
Cholesterol (mg/dL)	262	< 200
Triglycerides (mg/dL)	80	< 150
HDL (mg/dL)	64	> 40
LDL (mg/dL)	191	< 100

## Discussion

In this case report, we present a rare case of an elderly patient who was incidentally found to have MMD during her workup for stroke-like symptoms. The diagnosis of MMD in the elderly with multiple risk factors and comorbidities poses a challenge to vascular neurologists. Taking into account patient demographics, the typical presentations of MMD, its underlying pathology, as well as its hallmark angiographic findings, guides the diagnostic workup of MMD. In our patient of East Asian descent with no known past medical history, the presence of ICA and MCA stenosis, along with compensatory collateral network of vessels, was the key to diagnosing MMD. Patients with bilateral dilated and tortuous lenticulostriate and thalamostriate arteries forming the characteristic "puff of smoke" appearance with no known associated conditions are said to have MMD [8]. In contrast, patients with MMS have unilateral vascular pathology, and almost 30% of these patients eventually develop contralateral disease [9].

The management of MMD in elderly patients is unclear. It may be reasonable to pursue conservative treatment in asymptomatic or stable patients in the setting of multiple comorbidities and increased risk of postoperative complications. Classically, antiplatelet therapy is used to reduce the risk of ischemic events in MMD, whereby it is hypothesized to prevent embolism from forming microthrombi at the sites of arterial stenosis [10-12]. It has been argued that medical therapy alone does not halt the progression of the disease, even in asymptomatic patients [13]. Surgical revascularization remains the primary treatment of MMD [14], whereby untreated patients tend to progress over an average period of five years with poor outcomes [1]. Indeed, in a cohort of 39 patients, direct revascularization, including superficial temporal artery to middle cerebral artery bypass, resulted in a reduction of further ischemic events and was found to improve cerebral perfusion dynamics [15]. Similarly, indirect revascularization procedures such as pial synangiosis resulted in a reduction in the frequency of stroke events after surgery; among the 67.8% of the patients who had stroke events prior to surgery, 7.7% of them had strokes in the perioperative period within the first 30 days, and only 3.2% had stroke events within at least one year of follow-up [16]. Following a discussion with our patient and their family, a decision was made to pursue medical management only.

Very few descriptive studies and case reports analyzed moyamoya arteriopathy in the elderly population. In a series of 87 patients with MMD aged 50-67 yearsold with an average Suzuki stage 4-5, the authors found no significant differences in modified Rankin Scale (mRS) scores between surgically-treated patients and conservatively-treated patients [17]. Noteworthy, the presence of diabetes [17] and posterior circulation involvement [18], as well as an older age of onset, were found to be predictors of post-op adverse events. Gupta et al. reported that four of six patients with MDD aged between 60-71 years old who underwent surgical revascularization had either improved mRS scores or stable disease phenotype, whereas two patients on whom non-operative approaches were pursued had later suffered from intraparenchymal hemorrhages [19]. This latter case series suggests a possible beneficial role of surgical intervention in avoiding MMD-induced hemorrhage in the elderly population. Among patients with silent MMD, Kuroda et al. showed that even asymptomatic moyamoya arteriopathy may potentially cause ischemic or hemorrhagic stroke [20].

## Conclusions

Our report highlights that MMD is a highly heterogeneous disease where some patients remain asymptomatic with slow disease progression, whereas other patients present as early as five years of age. It is imperative to treat MMD early in the disease course to reduce the risk of further ischemic stroke events as well as permanent neurological deficits and neuropsychological sequelae. Further studies are warranted to investigate the role of medical and surgical approaches in elderly patients with moyamoya arteriopathy.
